# Selective Inhibition of Plasmodium falciparum ATPase 6 by Artemisinins and Identification of New Classes of Inhibitors after Expression in Yeast

**DOI:** 10.1128/aac.02079-21

**Published:** 2022-04-25

**Authors:** Catherine M. Moore, Jigang Wang, Qingsong Lin, Pedro Ferreira, Mitchell A. Avery, Khaled Elokely, Henry M. Staines, Sanjeev Krishna

**Affiliations:** a Institute for Infection and Immunity, St. George’s, University of London, London, United Kingdom; b Department of Biological Sciences, National University of Singapore, Singapore, Singapore; c Microbiology and Infection Research Domain, Life and Health Sciences Research Institute, School of Medicine, University of Minho, Campus de Gualtar, Braga, Portugal; d University of Mississippi, School of Pharmacy, Department of BioMolecular Sciences, Division of Medicinal Chemistry, Oxford, Mississippi, USA; e Institute for Computational Molecular Science, Temple University, Philadelphia, Pennsylvania, USA; f Department of Chemistry, Temple University, Philadelphia, Pennsylvania, USA; g St. George’s University Hospitals National Health Services Foundation Trust, London, United Kingdom; h Universitätsklinikum Tübingen, Tübingen, Germany

**Keywords:** *Saccharomyces cerevisiae*, antimalarial agents, drug discovery, drug resistance mechanisms, drug screening, mechanisms of action

## Abstract

Treatment failures with artemisinin combination therapies (ACTs) threaten global efforts to eradicate malaria. They highlight the importance of identifying drug targets and new inhibitors and of studying how existing antimalarial classes work. Here, we report the successful development of a heterologous expression-based compound-screening tool. The validated drug target Plasmodium falciparum ATPase 6 (PfATP6) and a mammalian orthologue (sarco/endoplasmic reticulum calcium ATPase 1a [SERCA1a]) were functionally expressed in Saccharomyces cerevisiae, providing a robust, sensitive, and specific screening tool. Whole-cell and *in vitro* assays consistently demonstrated inhibition and labeling of PfATP6 by artemisinins. Mutations in PfATP6 resulted in fitness costs that were ameliorated in the presence of artemisinin derivatives when studied in the yeast model. As previously hypothesized, PfATP6 is a target of artemisinins. Mammalian SERCA1a can be mutated to become more susceptible to artemisinins. The inexpensive, low-technology yeast screening platform has identified unrelated classes of druggable PfATP6 inhibitors. Resistance to artemisinins may depend on mechanisms that can concomitantly address multitargeting by artemisinins and fitness costs of mutations that reduce artemisinin susceptibility.

## INTRODUCTION

The rate of decline in global cases of malaria has diminished in recent years (1). Fortunately, artemisinin combination therapies (ACTs) continue to be effective in managing uncomplicated malaria, including in regions with multidrug-resistant parasites (2). Artemisinins in ACTs remain effective even when their partner drug is failing (3). In the past few years, decreased parasite clearance times following treatment with ACTs have been associated with decreased sensitivity of Plasmodium falciparum ring stages to dihydroartemisinin (DHA) (4). Prolonged parasite clearance times in and of themselves are not associated with treatment failures if the partner drug of an ACT is effective, but concerns have been raised about the risk of artemisinin resistance (5, 6).

Understanding how artemisinins act as antimalarials can help to optimize their use, increase insights into potential artemisinin resistance mechanisms, and help to develop them for other urgently needed indications, such as their repurposing as anticancer (7) or anti-severe acute respiratory syndrome coronavirus 2 (SARS-CoV-2) agents (8). Almost 2 decades ago, we suggested that artemisinins acted by inhibiting Plasmodium falciparum ATPase 6 (PfATP6), the sarco/endoplasmic reticulum calcium ATPase (SERCA) pump of malarial parasites, based on observed structural similarity between artemisinin and thapsigargin, a specific mammalian SERCA pump inhibitor (9). Several independent lines of evidence were consistent with this hypothesis, including the following observations. Synthesis of thaperoxide, which incorporates an endoperoxide bridge into thapsigargin, confirmed that these structures were relatable, because antimalarial potency and inhibition of PfATP6 were simultaneously increased (10). There was a positive correlation between inhibitor profiles for antimalarial action *in vitro* (in whole-cell assays) and after heterologous expression of PfATP6 (9). Artemisinin sensitivity in parasites transfected with the PfATP6 mutant ^L263E^PfATP6 showed increased variability in sensitivity assays, with decreased sensitivity to artemisinin and DHA in *ex vivo* and *in vivo* experiments (11). Fluorescent derivatives of artemisinin and thapsigargin colocalized in parasites (12). PfATP6 is an essential gene in parasites, as attempts to knock it out are lethal (13). Studies in unrelated systems, such as in mammalian cancer cell lines, have also demonstrated inhibition of SERCA by artemisinins (14, 15).

Heterologous expression of PfATP6 using *Xenopus* oocytes showed selective inhibition by artemisinins (9). Others attempted to reproduce inhibition by artemisinins using purified and reconstituted PfATP6 in membrane vesicles and after expression in oocytes but were unsuccessful (16).

Our understanding of the mechanisms of action of artemisinins has recently progressed through the use of click chemistry approaches. Artemisinins are alkylating agents that form covalent bonds (after activation) with their targets (17). This has allowed the labeling of dozens of proteins in asexual-stage P. falciparum and validation of some of them as potential targets of artemisinins. Two independent groups almost simultaneously identified several P. falciparum transporters by labeling with derivatized artemisinins. PfATP6 was included in this list of labeled proteins in both *in vivo* experiments (18, 19).

To advance these studies, we developed a Saccharomyces cerevisiae functional rescue assay using a codon-optimized construct of PfATP6. This robust assay allows screening and study of PfATP6 inhibitors and comparisons with mammalian SERCA, mutational analyses, biochemical studies, and scaling for higher-throughput investigations. We also translated findings from heterologous expression in yeast to studies in parasites to confirm their relevance, and we report findings using these approaches.

## RESULTS

### Screening tool development and optimization.

Saccharomyces cerevisiae K667 is hypersensitive to extracellular calcium because endogenous P-type calcium ATPases are inactive (calcium ATPase in yeast vacuole [PMC1]) or deleted (calcium ATPase in yeast Golgi [PMR1]). K667 is functionally rescued with heterologous P-type calcium ATPases, as described (13). To expand the repertoire available for screening inhibitors and mutated sequences, several modifications were introduced in expression studies.

**FIG 1:**
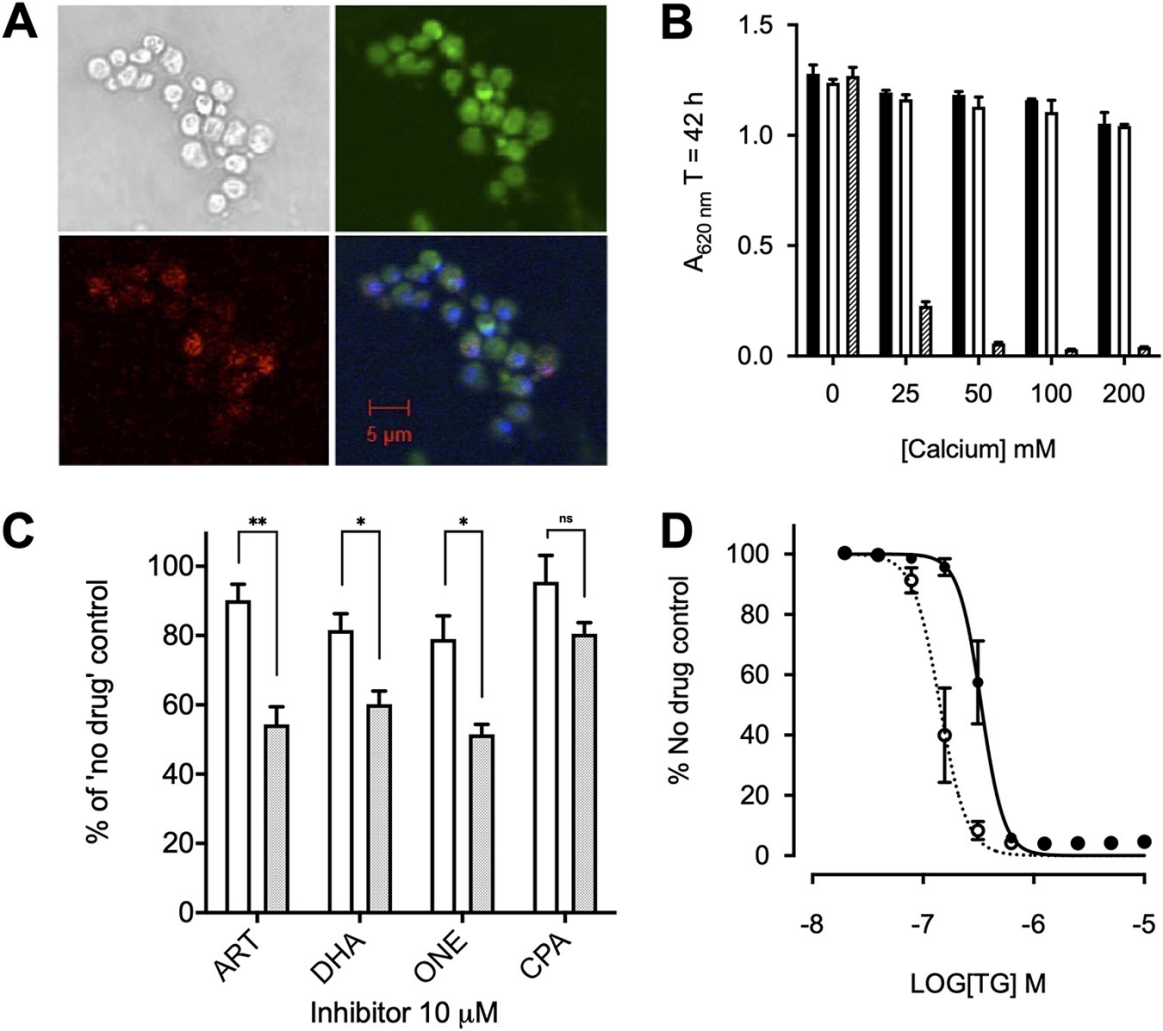
Yeast expression and optimization. (A) Immunofluorescence of SERCA1a. Yeast stained with primary SERCA1a antibody (Abcam) (top left), Texas Red-tagged goat anti-mouse IgG secondary antibody (bottom left), the ER-specific stain DiOC_6_(3) (top right), and the nucleic acid-specific stain DAPI (bottom right) are shown. (B) Growth of the K667[SERCA1a] strain (white), reference strain BY4741 (black), and K667[SERCA1a] treated with 1 μM thapsigargin (hatched) in an extracellular calcium concentration range. Error bars are SDs of the means of 5 technical replicates. (C) Growth inhibition comparison of the K667[SERCA1a] strain (white) and K667[PfATP6] strain (shaded) treated with 10 μM artemisinin (ART), DHA, artemisone (ONE), and CPA. Error bars are standard errors of the mean (SEMs) of 3 biological means of 5 technical replicates. Values were measured at 40 h. **, *P < *0.0075; *, *P < *0.05; ns, not significant. (D) Dose-response curves for the K667[SERCA1a] strain (solid line and black circles) and K667*Δpdr5*[SERCA1a] (dotted line and open circles) to thapsigargin. Values were measured at 40 h.

To compare with the mammalian SERCA1a pump, a new strain (*K667::SERCA1a*) was generated by transformation of K667 with plasmid pUGpd-SERCA1a. A vector-only control (*K667::pUGpd*) was included in experiments assessing the selectivity and specificity of inhibitors. SERCA1a expression in yeast (Fig. 1A) restores calcium tolerance (Fig. 1B) to levels similar to those seen by Pulcini et al. for PfATP6 (Fig. 2 in [13]). Sensitivity to inhibitors is more apparent when they are used to inhibit yeast growth in the presence of calcium, at concentrations approaching those maximally tolerated by a particular strain; total extracellular calcium concentrations of 20 to 40 mM are optimal for assessing the inhibition of PfATP6 (Fig. 3 in [13]). For SERCA1a, any concentration greater than 50 mM (up to 250 mM) is sufficient to monitor complete inhibition (see Fig. S1B in the supplemental material).

**FIG 2:**
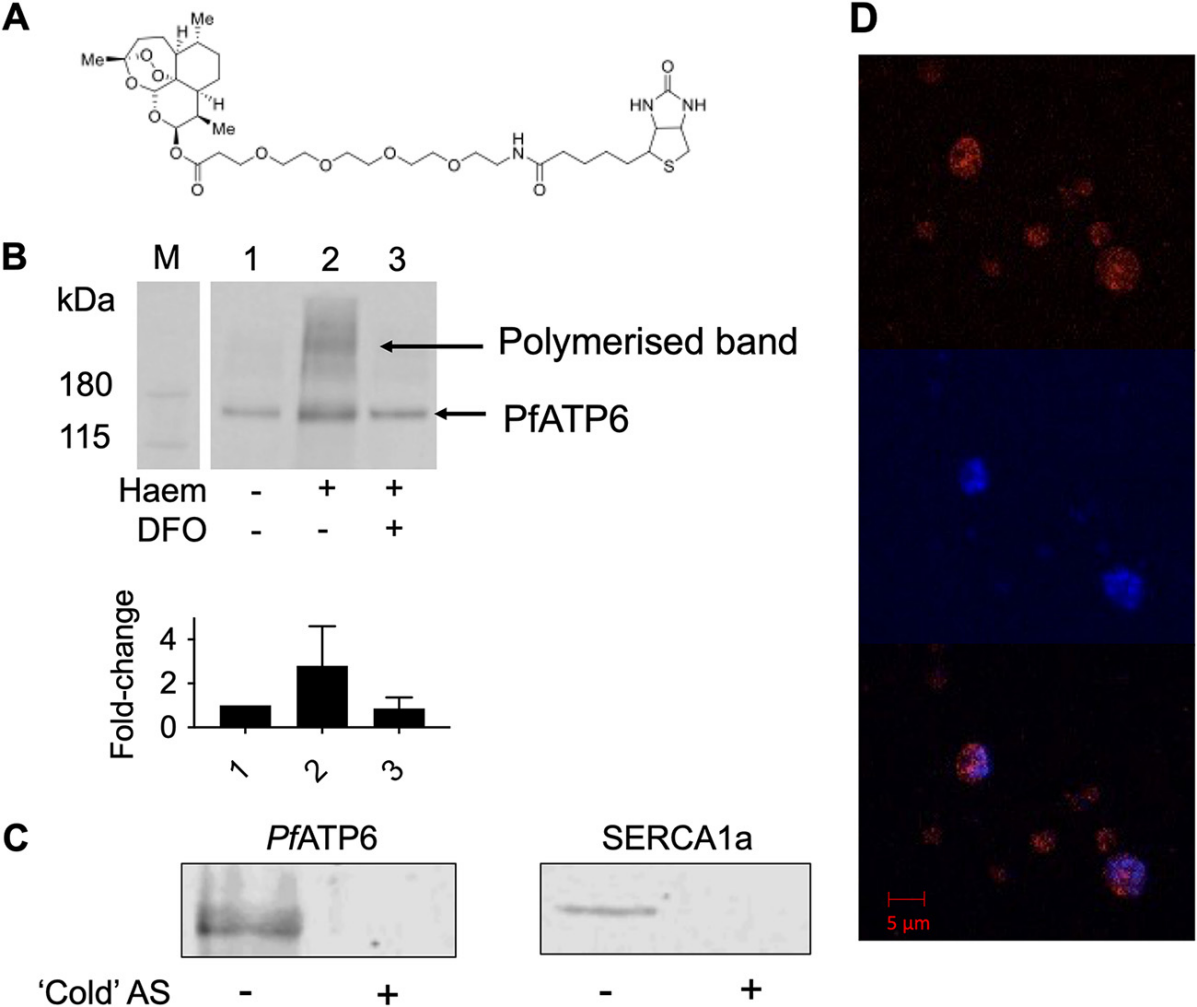
Artemisinins’ interaction with PfATP6. (A) DHA-biotin probe structure. (B) Western blots of PfATP6-enriched microsome pulldowns that were untreated (lane 1), preincubated with 200 μM heme (lane 2), or preincubated with heme and 200 μM DFO, which is an iron chelator (lane 3). Fold change refers to the band intensity relative to untreated PfATP6 (lane 1), calculated from densitometry. PfATP6-enriched microsomes were incubated with DHA-biotin before the addition of streptavidin-coated magnetic beads (Invitrogen, UK), which were pulled down using a magnetic rack. Beads were released by incubation in SDS at 95°C for 5 min. The supernatant was sampled for SDS-PAGE. (C) Western blots of PfATP6- and SERCA1a-enriched microsome pulldowns preincubated with artesunate (AS) before pulldowns were performed with DHA-biotin. (D) Immunofluorescence assay with P. falciparum parasites within red blood cells. Trophozoite-stage parasites were preincubated with DHA-biotin before staining with 1 μg/mL tetramethyl rhodamine isocyanate (TRITC)-tagged antibiotin antibody (red) (top) and the nucleic acid-specific stain DAPI (blue) (middle). (Bottom) Merge.

**FIG 3:**
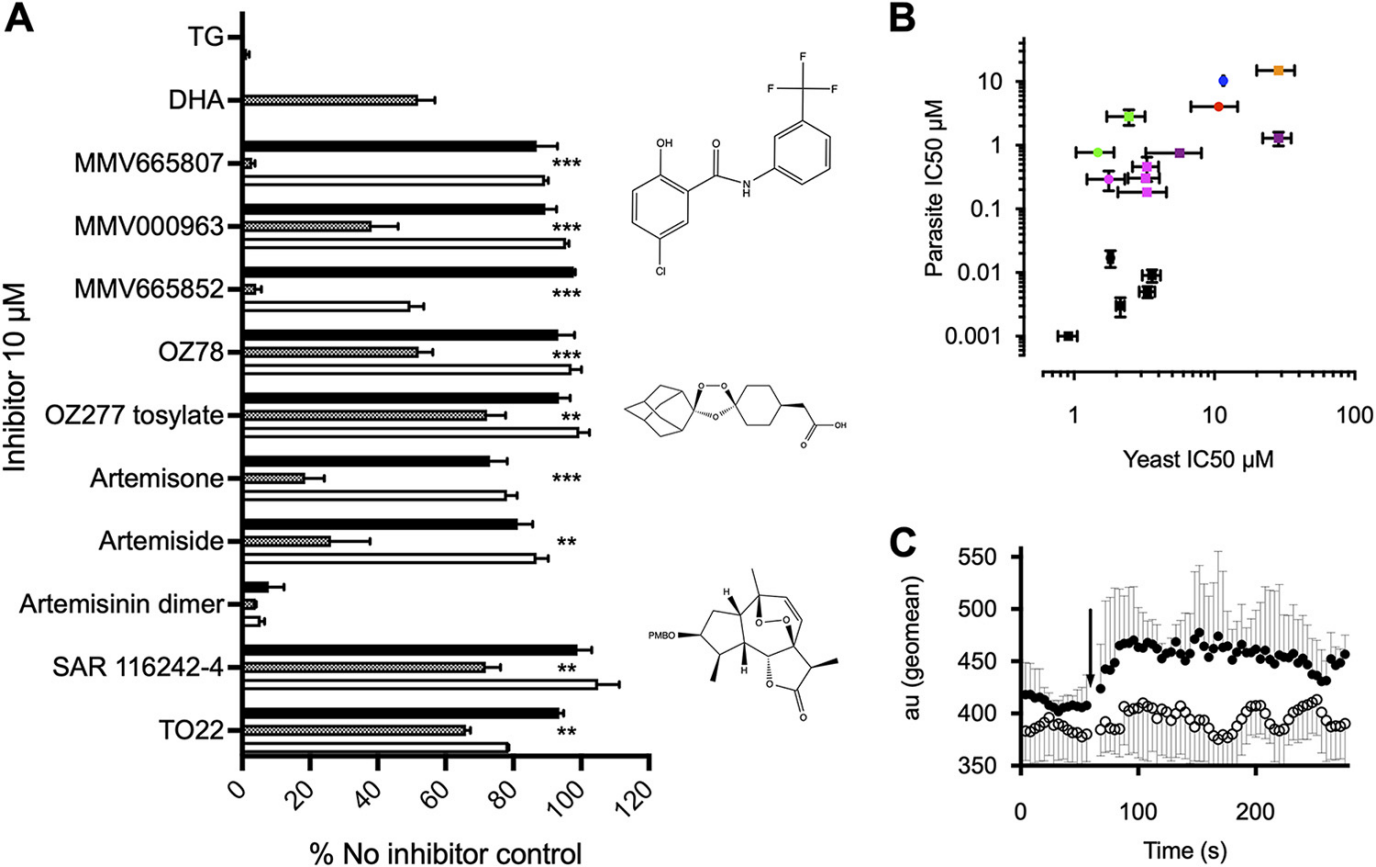
Inhibitor screens on whole yeast and parasites. (A) All compounds, including all 400 compounds in the malaria box, were screened against K667*Δpdr5*[PfATP6] (hatched bars) and two control strains, i.e., K667*Δpdr5*[SERCA1a] (white bars) and K667*Δpdr5*[pUG] (black bars), in a growth inhibition assay. Positive controls demonstrating inhibitor sensitivity were K667*Δpdr5*[SERCA1a] plus thapsigargin (TG) and K667*Δpdr5*[PfATP6] plus DHA. K667*Δpdr5*[pUG] was included in each inhibitor screen as a negative control. For each class of inhibitor, a representative structure is shown. Values were measured at 40 h. **, *P* < 0.0075; ***, *P* < 0.0001. (B) Correlation between the K667*Δpdr5*[PfATP6] yeast assay-derived IC_50_ values and the *in vitro* 3D7 Plasmodium falciparum assay-derived IC_50_ values for inhibitors, with an *r* value of 0.7 (Pearson analysis, *P = *0.004). Artemisinins are in black. MMV compounds are in green, magenta, red/orange, and purple. Circles denote parent compounds and squares derivatives. CPA is represented by the blue circle. Error bars are SEMs. (C) Changes in cytosolic Ca^2+^ concentration after application of artemisone. Shown is absorbance (mean ± SD of 3 independent replicates, in arbitrary units [au]) for YC-Nano-expressing parasites assayed in a BD LSR II flow cytometer, using 405-nm excitation and monitoring of the fluorescence resonance energy transfer (FRET) signal at 580 nm, with baseline tracings followed by addition (arrow) of artemisone (25 μM) (filled circles) or control (inactive derivative deoxyartemisone [25 μM]) (open circles). There is significant (*P < *0.001, multivariate ANOVA with repeated-measures analysis comparing control and experimental measurements) and sustained elevation in the cytosolic Ca^2+^ concentration after application of artemisone (traces being statistically comparable [*P > *0.05, multivariate ANOVA] before addition of inhibitor) but not the control compound. Initial experiments were blinded.

We confirmed that rescue in individual yeast strains was dependent on PfATP6 or SERCA1a by using cyclopiazonic acid (CPA) as a relatively unspecific inhibitor of both pumps. Artemisinin derivatives selectively inhibit PfATP6 function, leaving SERCA1a substantially less sensitive at comparable concentrations (Fig. 1C), and are used to establish specificity.

Yeast expresses ABC transporters that may modulate sensitivity to inhibitors in whole-cell screening assays by enhancing their efflux. We knocked out *PDR5*, a key efflux pump, to establish its contribution to sensitivity of whole-cell assays to inhibitors (20). The 50% inhibitory concentration (IC_50_) of thapsigargin in *K667DPDR5*::*SERCA1a* was >2-fold lower (141.8 ± 43.9 nM) than that in *K667*::*SERCA1a* (334.7 ± 67.5 nM [*P = *0.015]) (Fig. 1D). The IC_50_ values for different artemisinin derivatives in the *K667DPDR5*::*PfATP6* strain of yeast were 4 to 11 times lower than those in the PDR5-intact strain *K667*::*PfATP6* (Table 1). Therefore, *K667DPDR5* was used for subsequent experiments.

**TABLE 1:** IC_50_ values for each artemisinin derivative for K667[PfATP6] and K667*Δpdr5*[PfATP6]

Drug	IC_50_ (95% CI)^*a*^ (μM)		Fold difference^*b*^
	K667[PfATP6]	K667*Δpdr5*[PfATP6]	
Artemisinin	10.6 (8.2–13.8)	1.8 (1.3–2.6)	6^*c*^
Artesunate	14.8 (10.0–21.9)	3.7 (2.9–4.7)	4^*d*^
DHA	23.8 (16.9–33.7)	2.1 (1.6–2.8)	11^*c*^
Artemether	15.7 (12.5–19.7)	3.4 (2.8–4.1)	5^*e*^
Artemisone	3.6 (2.4–5.3)	0.9 (0.6–1.3)	4^*c*^

a CI, confidence interval.

b Significant differences between wild-type and *pdr5*-knockout strains for each derivative are indicated. Means are of three biological replicates.

c *P < *0.005.

d *P < *0.05.

e *P < *0.0005.

### Mechanism of action investigations.

Artemisinin’s mode of action is not yet fully elucidated, but it alkylates multiple proteins after being activated by heme in malarial parasites and in cancer cell lines (21). To confirm that PfATP6 is alkylated by artemisinins in yeast as well as parasites, we used a biotin-derivatized DHA (NewChem Technologies) (Fig. 2A) that retains antiparasitic potency (IC_50_ of 5.0 nM ± 2.0 nM, compared with 2.5 nM ± 1.4 nM for DHA [*P = *0.15]) to label PfATP6 or SERCA1a.

PfATP6 binding to DHA was confirmed through pulldown and Western blot analyses (Fig. 2B), and mass spectrometry identified several proteins alkylated by the tagged DHA when applied to membrane extracts and whole yeast, including PfATP6 in K667[PfATP6] preparations. Yeast lacking PfATP6 were not labeled at the masses shown in Fig. 2B (Table 2). SERCA1a could not be labeled to the same extent in parallel experiments (Fig. 2C), consistent with decreased potency of artemisinins against the native mammalian orthologue (Fig. 1C). Preincubation of PfATP6 with excess water-soluble artesunate competed with the tagged DHA, preventing it from binding (Fig. 2C). The addition of heme increased labeling of PfATP6 exposed to tagged DHA, as it does in parasites (Fig. 2B) (18). Conversely, chelation of Fe^3+^ using desferrioxamine (DFO) attenuates the interactions between artemisinins and targets (Fig. 2B). Immunofluorescence assays with parasites using a fluorescein isothiocyanate (FITC)-labeled antibiotin antibody localized the tagged DHA to the area surrounding the nucleus, most likely the endoplasmic reticulum (ER), which is where PfATP6 is localized (Fig. 2D) (13, 22).

**TABLE 2:** Mass spectrometry results of all proteins pulled down by DHA-biotin from both PfATP6-expressing whole yeast and PfATP6-enriched microsomes^*a*^

Unassigned proteins	Mitochondrial proteins	Proteins also identified in P. falciparum	Proteins also identified in yeast protein-enriched vesicles
Adh1p alcohol dehydrogenase Ahp1p Arc1p tRNA delivery Bgl2p endo-β-1,3-glucanase Bmh1p Bub2p Dur1,2p urea carboxylase/allophanate hydrolase Erg20p synthesizing Rer2p substrate farnesyl diphosphate Gag Nam9p Pdc1p peroxisomal protein of unknown function Rnr2p ribonucleotide-diphosphate reductase subunit RNR2 Rpl10p ribosomal, chaperone Rpl11ap Rpl16bp Rpl9ap Rps14ap Ubi4p ubiquitin binding Ugp1p UTP glucose-1-phosphate uridylyltransferase Uncharacterized protein Wtm1p transcriptional modulator	6-Phosphogluconate dehydrogenase Acc1p acetyl-coenzyme A carboxylase Acetyl-coenzyme A synthetase ATP synthase subunit α Cyc1p po-cytochrome *c* Cytochrome *c* oxidase subunit 2 (fragment) Glutamate decarboxylase Glyceraldehyde-3-phosphate dehydrogenase Malate dehydrogenase Pck1p phosphoenolpyruvate carboxykinase PCK1 Pet9p ADP/ATP transporter Phosphoglycerate kinase Por1p mitochondrial outer membrane protein of unknown function Pyruvate carboxylase Pyruvate kinase Qcr2p ubiquinol-cytochrome *c* reductase subunit 2 Succinate dehydrogenase Superoxide dismutase [Cu-Zn] Triosephosphate isomerase	40S ribosomal protein S21 40S ribosomal protein S81 **PfATP6** Act1p actin ATP-dependent 6-phosphofructokinase Elongation factor 1α Eno2p Eukaryotic translation initiation factor 5A Hsp104p Hsp12p Hsp26p Hsp30p Ribosomal protein L19 Rps18ap ribosomal subunit protein Ssa1p ATPase HSP70 family	**PfATP6** Arc1p tRNA delivery Elongation factor 1α Hsp30p Plasma membrane ATPase DNA-directed RNA polymerase I subunit RPA43 Rps11bp Sec28p coatomer subunit ε Yro2p

a Results are separated into unassigned proteins, mitochondrial proteins, proteins also pulled down from P. falciparum and human cancer cells in other studies, and proteins identified in yeast microsomes. The presence of PfATP6 is highlighted in bold.

### Compound screening.

Several individual compounds and three compound sets were screened using whole-cell assays: the Medicines for Malaria Venture (MMV) malaria box library (23), the MMV OZ box, and the thaperoxides (10). The MMV malaria box is a set of 400 compounds with (sub)micromolar antimalarial activity. Thaperoxides are derivatives of thapsigargin with an endoperoxide bridge introduced to resemble artemisinin more closely. The MMV OZ box is a blinded set of semisynthetic artemisinin derivatives. To define assay robustness, *Z*′ values were derived (24); the *Z*′ value for PfATP6 inhibition with CPA is 0.97 ± 0.123, and that for SERCA1a inhibition with thapsigargin is 1.00 ± 0.012.

Hits from the compound screen are summarized in Fig. 3A. Six hits were initially identified from the MMV malaria box. Three of these hits gave results that were too variable to take forward. MMV665807 showed the highest reproducible potency against PfATP6 with almost no inhibition of control strains (*K667Δpdr5*::*SERCA1a* and *K667Δpdr5*::*pUGpd*). Hits from the malaria box were characterized in more detail in *PDR5*-knockout strains and in cultured parasites. Identification of several new chemically unrelated classes of inhibitor of PfATP6 (Fig. 3B) confirmed a correlation between inhibitory constants for PfATP6 derived in yeast and antiparasitic potency (Pearson’s*r*^2^ = 0.7 [*n* = 3]; *P* = 0.004).

We next investigated whether addition of artemisone (the most potent PfATP6 inhibitor and antimalarial) perturbed calcium homeostasis by measuring the free Ca^2+^ concentration in parasites using a cameleon-Nano biosensor (25). Artemisone significantly increased the free Ca^2+^ concentration in parasites, confirming the relevance of inhibition of PfATP6 (Fig. 3C).

Other compounds suggested by the literature were screened but had limited success, including hypericin, benzohydroquinone (BHQ), saikosaponin, spiroindolones, and *tert*-butyl peroxide (see Fig. S1A) (26–29). Of these, saikosaponin and spiroindolones inhibited PfATP6 but only at high concentrations (>100 μM). These results are useful to train selection strategies for future compound screenings identifying PfATP6 inhibitors.

### Mutation effects on drug sensitivity.

**FIG 4:**
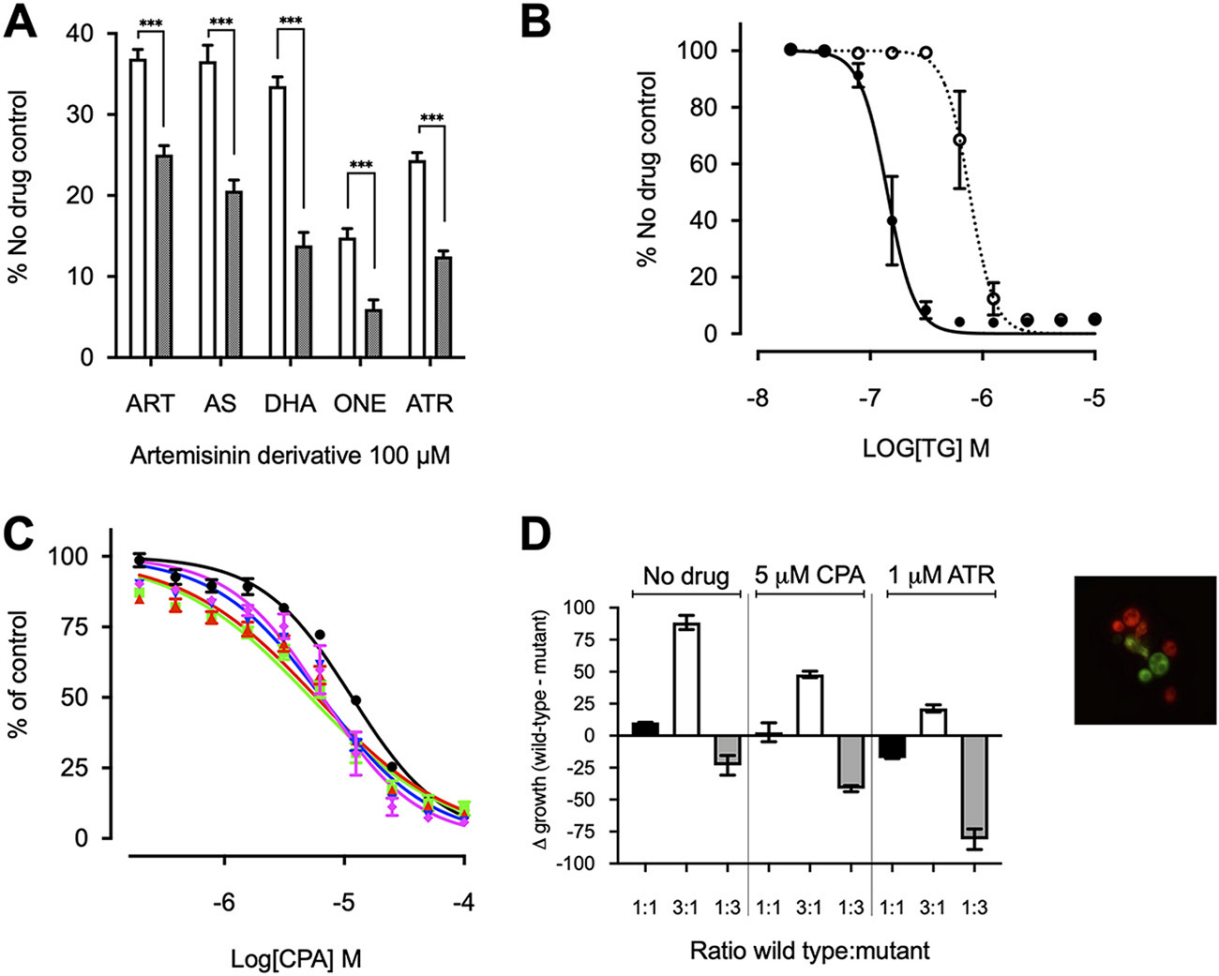
Effect of single-nucleotide polymorphisms on artemisinin sensitivity. (A) K667*Δpdr5*[SERCA1a]^E255^ (white) and K667*Δpdr5*[SERCA1a]^255L^ (black) were treated with 100 μM artemisinin (ART), artesunate (AS), DHA, artemisone (ONE), and artemether (ATR). ***, *P < *0.0001. (B) Dose-response curves with thapsigargin (TG) for K667*Δpdr5*[SERCA1a]^255L^ (dotted line) and K667*Δpdr5*[SERCA1a]^E255^ (solid line). Note that the IC_50_ for K667*Δpdr5*[SERCA1a]^E255^ is the same as that in Fig. 1D, because the assays were performed together. (C) Dose-response curves with CPA for wild-type K667[PfATP6] (black circles) versus strains expressing PfATP6 with resistance-conferring single-nucleotide polymorphisms S769N (blue triangle), L263E (green square), A623E (red triangle), and A623E/S769N (magenta diamond). (D) Fitness cost of K667*Δpdr5*[mCherry][PfATP6]^769N^, compared to the wild-type K667*Δpdr5*[Venus][PfATP6]^S769^, with and without drug pressure from 1 μM artemether (ATR) or 5 μM CPA. The extracellular calcium concentration was 50 mM. Fitness cost is presented as the change in growth between the wild-type and mutant strains after coincubation. Differences in growth (Δgrowth) (in percent) were calculated as the relative fluorescence of each strain when grown together at different starting ratios of wild-type strain/mutant strain, i.e., 1:1 (black), 3:1 (white), or 1:3 (gray). Means are of 2 biological replicates and 5 technical replicates. *P* values from Student’s *t* test comparing each ratio with and without drug were all <0.05.

After expression in *Xenopus* oocytes, sensitivity of SERCA1a to artemisinins was increased by mutating a key amino acid residue (E255L) in the thapsigargin binding pocket (30, 31). We confirmed that ^E255L^SERCA1a was more sensitive to all tested artemisinins and conversely became 5-fold less sensitive to thapsigargin, i.e., IC_50_ of 793.6 ± 247.4 nM versus 146.8 ± 43.9 nM for the wild-type strain (*P = *0.011) (Fig. 4A and B).

### Fitness cost.

Mutations in drug targets can have variable phenotypic manifestations in parasites due to opposing effects of decreased parasite fitness and decreased artemisinin sensitivity, as proposed previously (32, 33). To test this hypothesis, we introduced *in vitro* the artemether resistance-conferring mutations S769N and A623E in PfATP6, which were previously identified in field isolates (32, 34). These naturally occurring mutations were supplemented by an L263E mutation, which we previously showed to confer variable resistance to artemisinins in parasites, as it substitutes the malarial amino acid for the mammalian equivalent. Pulcini et al. (Table 1) (13) also showed that these mutations confer resistance to artemisinin and its derivatives in the yeast heterologous expression model. ^A623E^PfATP6 allowed the yeast to grow with all artemisinins at levels significantly higher than those for the wild-type strain, except with artesunate. Similar findings were noted for ^S769N^PfATP6 with DHA. Otherwise, all mutations improved yeast growth in the presence of artemisinins (13).

Mutated PfATP6 sequences were also assayed in yeast for sensitivity to CPA, which acts to inhibit SERCA pumps in different ways, both by interaction at different binding sites, compared with those predicted for artemisinins, and by hydrophobic interactions and not alkylation (35). All mutations (L263E, A623E, S769N, and A623E/S769N) increased sensitivity to CPA, both in parasites (13) and in the yeast heterologous expression assay (Fig. 4C). IC_50_ values (mean ± standard deviation [SD]) were 11.59 μM ± 0.02 for the wild-type strain versus 5.21 μM ± 0.03 for the L263 mutant, 5.86 μM ± 0.04 for the A623E mutant, 6.49 μM ± 0.03 for the S769N mutant, and 6.61 μM ± 0.041 for the A623E/S769N double mutant. There was a significant difference between the wild-type strain and all mutants (one-way analysis of variance [ANOVA] multiple comparisons, *P* < 0.0001). These findings are consistent with the suggestion that mutations of PfATP6 result in a fitness cost, as L263E also does in parasites (13). These changes in CPA sensitivity are unlikely to be binding site related because the CPA binding site is far from residue 263. It is more likely that the mutations studied cause impairment of the function of the pump. This fitness cost was also observed during assay optimization, where the yeast expressing mutant PfATP6 required a lower calcium concentration to grow optimally, compared to wild-type PfATP6.

### Fitness in yeast.

To examine the hypothesis that mutations in PfATP6 or SERCA1a decrease fitness in yeast rescue assays, we generated fluorescent reporter strains of yeast (Venus for wild-type PfATP6-expressing yeast and mCherry for the mutant PfATP6-expressing yeast) (see image box in Fig. 4D). These constructs were used in competition experiments between native and mutated PfATP6 sequences. Because ^S769N^PfATP6 was less sensitive to artemether in parasites in French Guyana, this mutation was selected for detailed investigation (32). Growth was unaffected by fluorescent markers, compared to the nonfluorescent congenic strain (see Fig. S1B), confirming that the markers do not cause a growth disadvantage.

Across a range of extracellular calcium concentrations (optimal concentration, 50 mM), native PfATP6 outcompeted ^S769N^PfATP6 in growth assays when experiments were begun at ratios of 1:1 or 3:1 (native strain/mutant strain) and continued until the yeast reached late log phase (18 h). If the two stains grew equally well, then the Δgrowth would be zero for 1:1 and 50 and −50 for 3:1 and 1:3, respectively. Growth levels were comparable when experiments began with a 1:3 ratio of the native strain to ^S769N^PfATP6. When selection with artemether (1 μM [approximately the yeast IC_80_]) was applied under these conditions, the fitness cost of ^S769N^PfATP6 became attenuated and yeast expressing this mutant had a growth advantage, especially when starting mixtures were in a ratio of 1:3 (native strain/mutant strain) (Fig. 4D). When CPA was used (5 μM [approximately the yeast IC_80_]) instead of artemether as a selective agent, ^S769N^PfATP6 lost this advantage.

**FIG 5:**
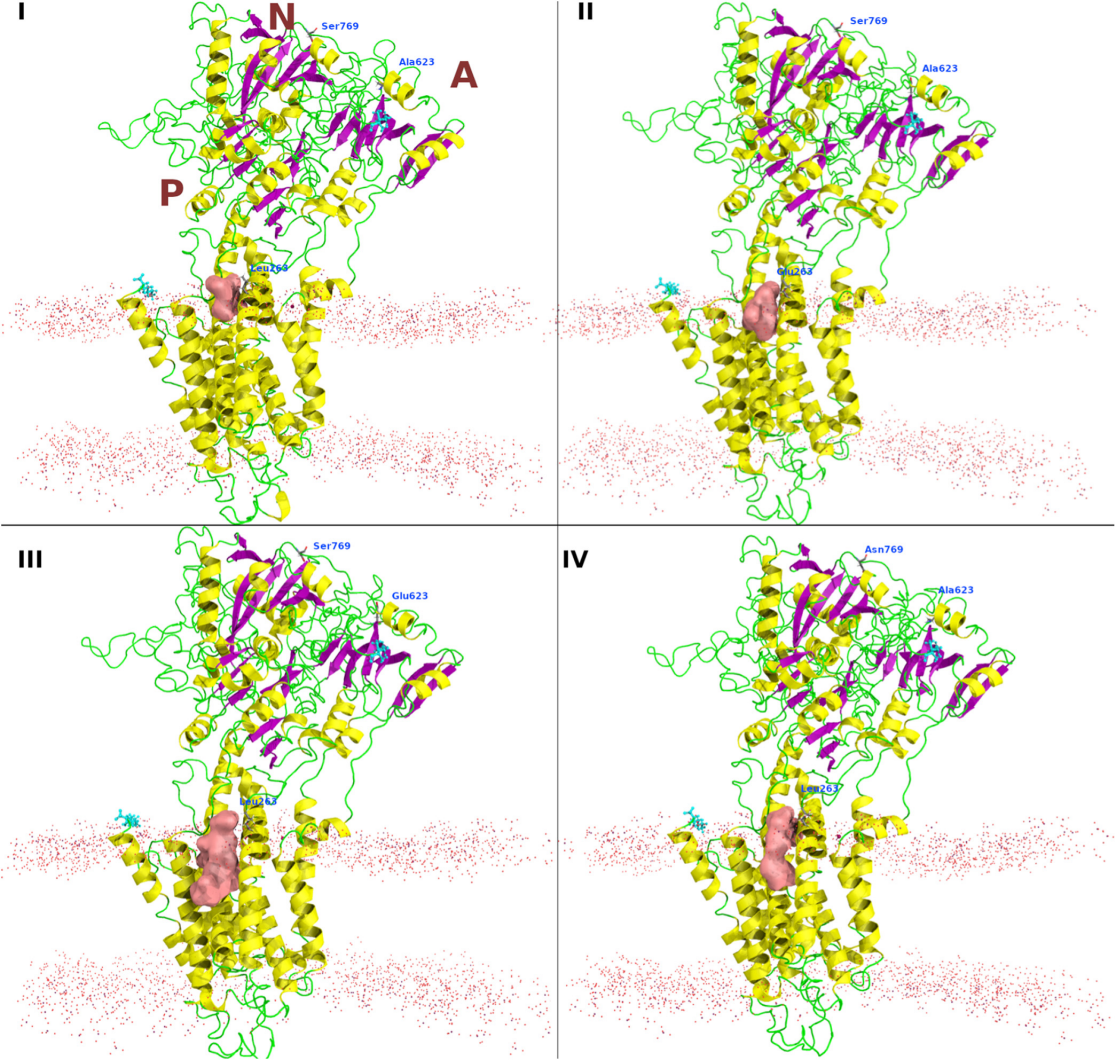
Homology models for the wild-type PfATP6 (I), ^L263E^PfATP6 (II), ^A623E^PfATP6 (III), and ^S769N^PfATP6 (IV). The druggable pocket close to Leu263 is shown as a reddish surface. The membrane position is shown as dots. The N and C termini are shown as cyan sticks. The cytosolic domains are labeled as N, A, and P, indicating the nucleotide-binding domain, the actuator domain, and the phosphorylation site, respectively.

*In silico* modeling shows the changes in the druggable pockets with different mutations (Fig. 5). The X-ray crystal structure of the SERCA in E2 (E309A mutant) was used as a template for building the homology models of PfATP6 and its mutants. The crystal structure shares 48% sequence identity with PfATP6, and it covers amino acids 5 through 1217. The predicted binding pocket for the modeled PfATP6, ^L263E^PfATP6, ^A623E^PfATP6, and ^S769N^PfATP6 has a volume of 624.411, 922.372, 1,746.135, and 1,048.841 Å^3,^ respectively. The mutations, even the distant ones, led to changes in the volume of the binding cavity adjacent to Leu263.

## DISCUSSION

More than 3 decades ago, we suggested that parasite integral membrane transport proteins may be useful drug targets (36, 37). In the course of investigating various families of transport proteins, including putative P-type cation ATPases we first isolated, we first functionally characterized a hexose transporter in *Xenopus* oocytes to establish a heterologous expression system. We then genetically validated the parasite’s hexose transporter, Plasmodium falciparum hexose transporter (PfHT), as a new drug target (38). Cocrystallization of PfHT with the selective inhibitor we characterized has confirmed that structure-based design of inhibitors against integral membrane proteins of P. falciparum is feasible (39).

Our early work also pointed to a family of P-type cation ATPases as being promising for further study as drug targets. PfATP4 was characterized as an atypical cation ATPase that was initially proposed to be a calcium ATPase (40) in oocytes and then was found to export sodium from the intraerythrocytic parasite (41). These studies are particularly interesting because PfATP4 is now an established drug target for several novel classes of antimalarial drugs, including the spiroindolones, which have reached clinical studies in development (42). PfATP4 also has mutations that can confer resistance to inhibitors and drugs (43).

PfATP6 has also been confirmed as a validated drug target by genetic studies (13). Artemisinins were first shown to inhibit PfATP6 after expression in *Xenopus* oocytes and to interact with PfATP6 in parasites (9). These observations led to the hypothesis that PfATP6 was the key target of artemisinins’ antiparasitic activity. Since then, several studies have shown that artemisinins display promiscuity in their targeting of parasite proteins by alkylating them (18, 19). This mechanism may explain several features of assays with artemisinins. For example, parasites display both greater variances and inconsistencies in IC_50_ values for artemisinins when bearing mutations in PfATP6 (11) predicted to reduce the susceptibility to artemisinins. The presence of more than one important target for artemisinins’ antimalarial activity (64 to 123 artemisinin-labeled proteins were identified, although not all would be plausible targets) may explain some variability in IC_50_ results, because secondary or tertiary targets may become more important if a primary target becomes less susceptible. This suggestion is reassuring regarding the risk of developing artemisinin resistance, because multiple targets may maintain parasite sensitivity to this class of antimalarial. Consistent with this suggestion of multiple targets, there may even be more than one binding site within a single protein target.

Different artemisinin derivatives behave differently against mutant PfATP6 *in vitro*. Adding to this complexity in interpreting susceptibilities of parasites with mutations in PfATP6 is their fitness cost, suggested in cultured parasites for those bearing ^S769N^PfATP6 and now demonstrated in yeast models (33). It also explains why it might have proved difficult to establish parasites with some PfATP6 mutations in cultures *ex vivo* for longer-term further study. The effects of mutations in PfATP6 observed in field isolates and in laboratory models of parasites and after heterologous expression are consistent with these mutations being able to exert remote influence on the properties of drug-binding regions (Fig. 5). Expression in yeast suggests that mutations in the cytosolic domains of PfATP6 (S769N and A623E, for example) can also exact fitness costs, and these costs may be ameliorated under drug selection pressure with some artemisinin derivatives. Figure 4C reproduces properties of increased susceptibility to CPA with ^S769N^PfATP6 that we observed with ^L263E^PfATP6 (13). The fitness competition assays with selection under CPA pressure (Fig. 4D) do not discriminate between mutant and wild-type PfATP6 sequence-bearing yeast strains, perhaps because CPA is a reversible inhibitor, unlike artemether, or because of some other undefined differences in the assay systems in which yeast strains are mixed.

To address differences in the results of assays of PfATP6 in *Xenopus* oocytes reported by different groups, we developed a robust yeast whole-cell assay to study artemisinins, to compare results to those with mammalian SERCA, and to allow screening of inhibitors. Earlier immunolocalization experiments demonstrated that PfATP6 is expressed in internal membrane structures in yeast (13). The alkylation of PfATP6 by an artemisinin derivative when expressed in yeast (as in parasites), together with growth rescue of inhibition by artemisinins in this model, confirms our earlier observations made in *Xenopus* oocytes on inhibition of PfATP6 and the effects of mutations (9, 30).

To confirm that mammalian SERCA1a can be made more susceptible to artemisinins, we mutated its sequence as before (^E255L^SERCA1a) and again demonstrated increased susceptibility to artemisinins (30) (Fig. 4A). Interestingly, this sequence is less susceptible to thapsigargin (Fig. 4B). These observations confirm that artemisinins target PfATP6 and that this inhibition can result in increases in free intraparasitic calcium concentrations when challenged with a potent artemisinin (Fig. 3C). These observations also highlight how methodologies used to assay function in different expression systems may give apparently inconsistent results. *In vitro* activity assays also demonstrate this point, as no compound tested showed any inhibition of purified PfATP6 despite apparent inhibition of purified SERCA1a by CPA in parallel experiments, suggesting that PfATP6 may not function in a representative way in *in vitro* assays (see Fig. S1C and D in the supplemental material).

Treatment failure with ACT emerged in the early 1990s and is currently localized to the Greater Mekong region (44, 45). Fortunately, appropriately selected ACTs continue to be effective in treating P. falciparum infections in this region when the partner drug is efficacious, and case numbers continue to fall (46).

Using PfATP6 as a model target allowed assessment of the importance of other targets, because, even with mutations that are predicted to decrease the sensitivity of PfATP6, assays in parasites bearing such mutations suggested more variable IC_50_ results, with both field and laboratory-generated strains. Fitness cost in the yeast model is ameliorated when these PfATP6 mutant strains are exposed to artemisinin derivatives, perhaps explaining why some parasite strains have been unstable in the laboratory when grown without drug selection pressure (33). Future fitness cost assays could be carried out with yeast strains that have fluorescent markers incorporated into chromosomal DNA, rather than in a vector, to provide a simpler “plug-and-play” platform.

To address problems of drug resistance in parasites, we exploited the yeast screening model because it is eukaryotic and easily genetically manipulated and knockout libraries are freely available (e.g., through EUROSCARF, Germany). One limitation is that any proposed target needs to have an orthologue in yeast; therefore, targeting mechanisms that relate to parasite-specific properties such as red cell invasion might be difficult. Also, antiparasitic drugs are often stage specific (which needs to be taken into account when choosing targets, since yeast do not have comparable life cycles). Yeast can respire aerobically or anaerobically, depending on the carbon source in the medium, further distinguishing it from parasites, and an inhibitory effect on yeast aerobic respiration apparatuses is apparent only when the carbon source ensures that yeast rely on mitochondrial respiration, rather than glycolysis (47).

Inhibitory constants in yeast screens were correlated with those in parasite assays. Differences in their magnitude (e.g., artemisinin value of 17 nM in parasites versus 1.8 μM in yeast) could be explained by several factors. A requirement for activation of artemisinins by heme is a possibility, because heme was not included in the yeast growth assays. Heme abolishes artemisinins’ ability to inhibit yeast growth in fermentable media (47). There might also be off-target effects in a whole-cell yeast system, such as pH perturbations, although these do not interfere with rescue by mammalian SERCA in our experimental system. Also, yeast has many efflux pumps that decrease its sensitivity to inhibitors, and *K667DPDR5*::*HIS[PfATP6]* yeast was 4 to 11 times more sensitive to artemisinins than the K667[PfATP6] strain. We did not knock out other potential efflux pumps, of which more than a dozen remain. This mechanism, as well as its relevance to parasites, is amenable to further study by introduction of, for example, the P. falciparum efflux pump PfMDR1.

In conclusion, several independent experimental findings are consistent with the hypothesis that artemisinins target PfATP6. PfATP6 can be inhibited by a variety of unrelated compounds after heterologous expression in a yeast screening system, demonstrating its potential as a drug target, and potencies correlate with parasite killing. Mutations in PfATP6 can incur fitness costs. Because artemisinins interact with multiple targets, including PfATP6, our findings suggest that decreased sensitivity to artemisinins (assayed in ring stages) may depend on a global response of the parasite by selection to delay its developmental cycle. The findings also suggest that the term “artemisinin resistance” may be best reserved for parasites that have been selected to demonstrate increased IC_50_ values for artemisinins in conventional *in vitro* assays, rather than those focused on decreased ring stage sensitivity.

## MATERIALS AND METHODS

### Reagents.

All reagents were purchased from Sigma-Aldrich except for thaperoxides (made by M. Avery). The OZ derivatives and the malaria box compounds were kindly donated by the MMV (23). The reference strain and K667 strain were from EUROSCARF (Germany), and the plasmid containing SERCA1a was kindly donated by Michel Ghislain (Université Catholique de Louvain, Louvain-la-Neuve, Belgium). Plasmids with fluorescent markers were kindly donated by Elizabeth Bilsland (Cambridge Systems Biology Centre and Department of Biochemistry, University of Cambridge, Sanger Building, Cambridge) (20). All kits were purchased from Qiagen. All mass spectrometry was performed by Jigang Wang. Antibodies were purchased from Abcam.

### Yeast strains.

The reference strain used was BY4741 (*MAT***a** *his3*Δ*1 leu2*Δ*0 met15*Δ*0 ura3*Δ*0)*. The calcium ATPase-knockout stain used was K667, from parental strain W-303 (*MAT***a** *ade2*Δ*1 can1*Δ*100 his3*Δ*11*,*15 leu2*Δ*3,11*,*2 trp1*Δ*1 ura3*Δ*1 cnb1*::*LEU1 pmc1*::*TRP1 vcx1*Δ).

### Transformations.

Yeast transformations were performed using the method described by Gietz and Schiestl (48), and transformants were selected by an uracil auxotrophic marker. Transformations were confirmed by colony PCR. Both K667 and *K667Δpdr5::HIS* were transformed with the following plasmids: pUGpd plasmid as a vector-only control (derived from pRS316 [49]); br434 plasmid (derived from pRS316 [50]) containing the SERCA1a coding region from rabbit skeletal fast-twitch muscle (*pbr434-SERCA1a*); and pUGpd containing the yeast-optimized coding region of PfATP6 from Plasmodium falciparum (*pUGpd-PfATP6*). Site-directed mutagenesis was performed on the SERCA1a coding region to produce the mutation E255L in the protein. Mutations were also introduced into the PfATP6 coding region to produce mutations L263E, A623E, and S769N and the double mutation A623E/S769N (13).

### Homologous recombination.

To knock out the *PDR5* gene from the K667 strain of yeast, the auxotrophic marker HISMX was amplified using the primers (20) 5′-AGACCCTTTTAAGTTTTCGTATCCGCTCGTTCGAAAGACTTTAGAATGGCAGAACCAGCC-3′ and 5′-TGTTTATTAAAAAAGTCCATCTTGGTAAGTTTCTTTTCTTAACCATACTTCACATCAAAA-3′, where the underlined region is homologous with HISMX. PCR products were used to transform the K667 strain as above. Knockouts were selected by their ability to grow on histidine-depleted selective medium. Recombination was confirmed with colony PCR.

### Immunocytochemistry.

Immunocytochemistry was performed as described by Kilmartin and Adams (51). Briefly, yeast were grown to an optical density (OD) equivalent to log phase and were prepared for immunostaining by removing the cell wall. The cell suspension was spotted on polylysine-coated microscope slides, dried, fixed, and permeabilized (10-min incubation in phosphate-buffered saline [PBS] with 0.1% Triton X-100); 1 μg/mL primary antibody (mouse anti-SERCA1a; Abcam) was added and incubated overnight at 4°C, and then 1 μg/mL secondary antibody (Texas Red-tagged goat anti-mouse IgG) was added and incubated for at least 2 h. The slides were stained with 5 μM 3,3′-dihexyloxacarbocyanine [DiOC_6_(3)] for 20 min to stain the ER. Antifade mounting medium plus 4′,6-diamidino-2-phenylindole (DAPI) was added before the slides were sealed. Yeast were visualized with a Zeiss confocal LSM 510 microscope.

Immunofluorescence with P. falciparum parasites was performed as described by Pulcini et al. (13), using the paraformaldehyde/glutaraldehyde fixation method. Parasites were labeled as described by Wang et al. (18), using 500 nM DHA-biotin probe. Parasites were visualized with a Zeiss confocal LSM 510 microscope.

### Site-directed mutagenesis.

The E255L mutation was introduced into SERCA1a using the following primers: forward, 5′-GCCGCTGCAGCAGAAGCTGGATTTATTCGGGGAGCAG-3′; reverse, 5′-CTGCTCCCCGAATAAATCCAGCTTCTGCTGCAGCGGC-3′. Site-directed mutagenesis was performed using the Agilent Quikchange Lightning site-directed mutagenesis kit, following the manufacturer’s instructions. Mutants were screened by Sanger sequencing (Eurofins, UK).

### Whole-cell screening assay.

The screening assay used here was developed from the protocol used by Pulcini et al. (13). A single colony of the yeast strain to be screened was picked and grown in 5 mL of selective medium until stationary phase was reached. The culture was diluted 100-fold in ampicillin-supplemented yeast extract-peptone-dextrose (YPD) medium to an OD at 620 nm (OD_620_) equivalent to that of lag phase. YPD medium was supplemented with an appropriate concentration of calcium, depending on the transformed strain. The optimal calcium concentration for the PfATP6-expressing strain was 22 mM, and that for the SERCA1a-expressing strain was 100 mM. The optimal calcium concentration was periodically monitored and, if necessary, adjusted using a calcium concentration range of 10 to 30 mM. A final volume of 200 μL of the yeast culture was added to each well of a 96-well plate, with 5 technical replicates included per inhibitor. All inhibitors were dissolved in dimethyl sulfoxide (DMSO) unless otherwise stated. Stock solutions of each inhibitor were diluted 100-fold in each well to give the desired final concentration. Where IC_50_s were being determined, inhibitors were 2-fold titrated from stock solutions. The 96-well plates were incubated at 30°C for 42 h. The yeast growth was estimated from the absorbance at 620 nm in a Tecan plate reader. Growth was normalized with the no-drug control to present data as percent growth. The data collected were analyzed using GraphPad Prism v9.0 (GraphPad software, San Diego, CA, U.S.A).

### *In vitro* parasite assays.

Inhibitor IC_50_s were calculated in parasite assays following the protocol described by Desjardins et al. (52). Briefly, cultures were synchronized to ring stage by D-sorbitol lysis, using a 5% D-sorbitol solution. The parasites were diluted to a final parasitemia of 1%. Hypoxanthine-free medium was added to give a hematocrit level of 4%. A 2-fold dilution series of the inhibitors in hypoxanthine-free medium was prepared, and 100 μL of each inhibitor concentration was seeded in a 96-well plate, with 5 technical replicates of each included. Two no-inhibitor controls, i.e., hypoxanthine-free medium only, were included. Plates were incubated at 37°C in 5% CO_2_ for 24 h. After 24 h, [^3^H]hypoxanthine was added to each well to a final concentration of 0.5 μCi/well, and the plates were incubated for a further 24 h. Plates were then freeze-thawed to lyse the cells, and the [^3^H]hypoxanthine uptake was measured in a Beckman scintillation counter.

Parasite calcium homeostasis was measured as described by Pandey et al. (25), using 25 μM artemisinin derivative artemisone, and compared to results with the inactive derivative deoxyartemisone. Three biological replicates were carried out, after an initial blinded experiment.

### Fitness cost assays.

Fitness cost assays were performed in yeast by transforming the strain K667[PfATP6]^wt^ with yEpGAP-Venus and the strain K667[PfATP6]^769N^ with yEpGAP-Cherry. Both vectors were kindly donated by Elizabeth Bilsland (20). Growth of the transformed and untransformed strains was compared using growth curves, performed as described by Moore et al. (47). Standard curves were generated for each strain by measuring fluorescence at each OD in a 2-fold dilution series of the yeast. The yeast were diluted to an OD equivalent to log phase and coinoculated at equal starting ODs. Yeast were incubated at 30°C for 24 h, at which point the yeast were in late log phase. The fluorescence was then measured for both fluorophores, and growth was estimated by calculating the OD using the standard curve. The OD was also measured. The experiments were repeated in the presence of drug pressure using 1 μM artemether or 5 μM CPA (i.e., approximate IC_10_).

Standard curves calculated from OD measurements and fluorescence measurements of titrations of each fluorescent strain of yeast were used to relate relative fluorescence to the equivalent OD, such that each strain in a 1:1 mixture should have equivalent relative fluorescence and make up 50% each of the total OD.

### *In silico* modeling.

Prime was used to construct and refine the three-dimensional (3D) models of wild-type PfATP6, ^L263E^PfATP6, ^A623E^PfATP6, and ^S769N^PfATP6 (the template has PDB accession code 5ZMV). The BLAST homology search was used to identify the most homologous protein structures from the PDB repository (http://www.rcsb.org) by using position-specific iterative BLAST, the NCBI nonredundant database, the BLOSUM62 similarity matrix, a gap opening cost of 11, a gap extension penalty of 1, an inclusion threshold of 0.005, and three iterations. The secondary structure prediction was then established by SSPro, followed by sequence alignment with ClustalW. Knowledge-based 3D model building was used to construct the models for each target sequence. Loops were refined by using the VSGB solvation model and extended serial loop sampling. Side chains were then repredicted for the refined loops, and the final 3D models was energy minimized using an OPLSe force field and VSGB solvation model. The protein structure quality was checked in the Schrӧdinger suite. The binding pockets of PfATP6 and all mutations were predicted using f-pocket to identify the most plausible cavities close to L/E263.

### Microsome preparation.

Protein-enriched microsomes were prepared as described by Nakanishi et al. (53) and Hwang et al. (54). Briefly, yeast were grown in 1 L of YPD medium until an OD_620_ of 1.5 to 2.0 was reached. Yeast were pelleted and resuspended in 50 mL of buffer 1 (0.1 M Tris-HCl [pH 9.4], 50 mM 2-mercaptoethanol, 0.1 M D-glucose) per 500 mL culture. After shaking at 30°C for 10 min, the yeast were pelleted, resuspended in 50 mL of buffer 2 (0.9 M D-sorbitol, 0.1 M D-glucose, 50 mM Tris-morpholineethanesulfonic acid [MES] [pH 7.6], 5 mM dithiothreitol [DTT], 0.5× selective defined medium, 0.05% [wt/vol] Zymolase 20T) per 500 mL original culture, and incubated at 30°C for 1 to 2 h, with gentle shaking. The yeast were pelleted and washed with 30 mL of 1 M sorbitol per original culture. The yeast were pelleted, resuspended in 20 mL of buffer 3 (50 mM Tris-MES [pH 7.6], 1.1 M glycerol, 1.5% [wt/vol] polyvinylpyrrolidone with an average molecular weight of 40,000, 5 mM EGTA, 1 mM DTT, 0.2% [wt/vol] bovine serum albumin [BSA], 1 mM phenylmethylsulfonyl fluoride [PMSF], 1× protease inhibitor) per original culture on ice, and homogenized. The homogenate was pelleted, and the supernatant was transferred to ultracentrifuge tubes. The pellet was resuspended in buffer 3 and pelleted again. This second supernatant was pooled with the previous and centrifuged at 150,000 × *g* for 45 min at 4°C. The pellet was resuspended in 1 to 2 mL buffer 5 (5 mM Tris-MES [pH 7.6], 0.3 M D-sorbitol, 1 mM DTT, 1 mM PMSF, 1× protease inhibitor) and aliquoted for single use before freezing in liquid N_2_.

### Mass spectrometry and activity assays.

Both whole yeast and microsomes were prepared for mass spectrometric analysis by addition of 500 nM biotin-tagged DHA probe to 200 μL (∼0.6 mg protein) of yeast microsome preparation or 10 μM probe to 50 to 100 mL yeast culture at an OD_620_ of 1.0 to 1.5 (∼35 to 70 mg total protein) and incubation for 4 h at 30°C. For competition studies, 25× artesunate was added and incubated for 30 min before addition of the probe and incubation for 4 h at 30°C. Samples were then acetone precipitated by adding 4 volumes of −20°C acetone/water (4:1) to the yeast pellet or microsomal pellet and incubating the mixture for 2 h to overnight at −20°C. The samples were then pelleted at 13,000 to 16,000 × *g* for 10 min at 0°C to 4°C and washed twice with −20°C acetone/water solution. After a final centrifugation, pellets were dried in a freeze-drier for 1 h. Subsequent pulldowns and liquid chromatography-tandem mass spectrometry were performed as described by Wang et al. (18).

Activity assays were performed as described by Longland et al. (55) and LeBel et al. (56), except that the reaction mixtures were incubated at 30°C instead of 37°C. Briefly, microsomes were diluted to 75 mg/mL, with a free calcium concentration of approximately 1 mM, in 2 mL assay buffer (45 mM HEPES/KOH [pH 7.2], 6 mM MgCl_2_, 2 mM NaN_3_, 250 mM D-sucrose, 12.5 mg/mL A23187, 2 mM EGTA, with or without 2 mM CaCl_2_) and incubated for 10 min at 30°C in presence or absence of inhibitor (10 to 100 μM). Then, 5.8 mM ATP was added and incubated for an additional 15 min at 30°C. The reaction was stopped with 0.5 mL 6.5% trichloroacetic acid (TCA) on ice, and the mixture was centrifuged at 4°C for 10 min at 16,000 × *g*; 0.5 mL of the supernatant was added to 1.5 mL of copper acetate buffer (0.25% copper sulfate pentahydrate, 4.6% sodium acetate trihydrate, dissolved in 2 M acetic acid [pH 4.0]) and mixed by vortex-mixing before the addition of 0.25 mL of 5% ammonium molybdate, followed by 0.25 mL METOL buffer (2% *p*-methyl-aminophenol sulfate, 5% sodium sulfite). Samples were incubated for 10 min, and the absorbance was measured at 870 nm in a spectrophotometer. The P_i_ standard was 0.4387 g/100 mL KH_2_PO_4_.

### Pulldown assays.

A 0.3-mg aliquot of PfATP6-enriched membrane vesicles, as well as an aliquot of SERCA1a and vector-only membrane vesicles, were thawed on ice. One-half of each aliquot (0.15 mg) was preincubated with 12.5 μM artesunate at 30°C for 30 min. Then, all samples had 500 nM DHA-biotin probe added (NewChem Technologies) and were incubated at 30°C for 4 h. The samples were then pulled down using Dynabeads, following the manufacturer’s instructions; 2 mg of beads was used per sample. Beads and samples were boiled for 5 min in 0.1% SDS to separate the beads from the proteins, and supernatants were run on a polyacrylamide gel. Western blotting was performed according to the NuPage Western blot protocol using 10% Bis-Tris gels (Thermo Fisher Scientific, UK) with mouse anti-SERCA1a or goat anti-PfATP6 primary antibody (1:1000) and LiCor fluorescently tagged donkey anti-mouse IgG or donkey anti-goat IgG secondary antibodies (1:10,000). Blots were analyzed using the Odyssey scanner system. Densitometry was performed using Image Studio software.

### Statistics.

Unpaired *t* tests and one-way ANOVA were performed using GraphPad Prism software.
