# Supplementary material for: Selective Inhibition of Plasmodium falciparum ATPase 6 by Artemisinins and Identification of New Classes of Inhibitors after Expression in Yeast
Source: Antimicrob Agents Chemother. 2022 Apr 25;66(5):e02079-21. doi: 10.1128/aac.02079-21 (PMC9112895; doi:10.1128/aac.02079-21)
Supplement: Supplemental file 1 — Fig. S1. Download aac.02079-21-s0001.pdf, PDF file, 0.1 MB [file aac.02079-21-s0001.pdf]

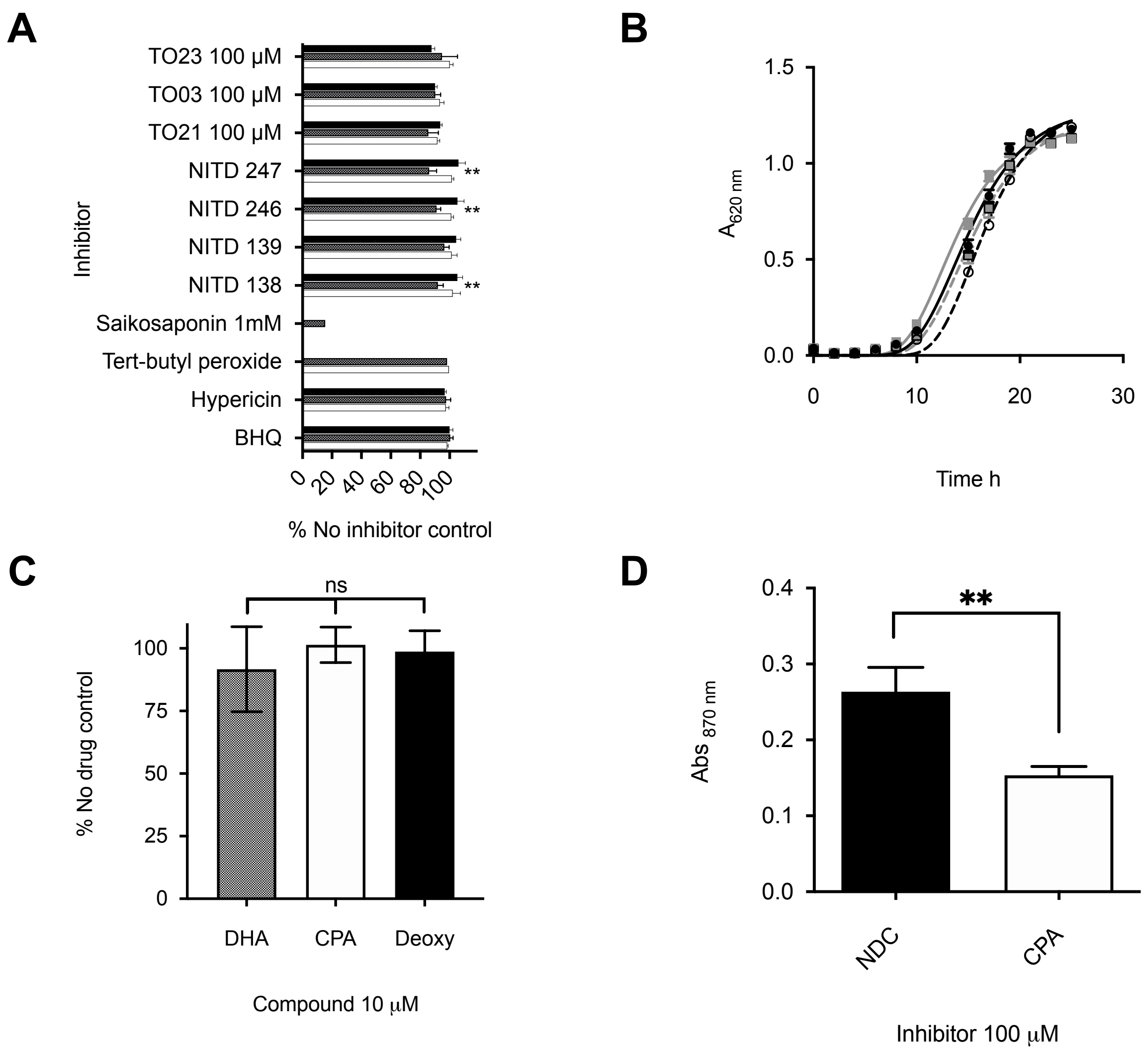


Supplementary Figure 1. **A.** Compounds which did not inhibit the target, except at high concentrations. Unless stated otherwise, inhibitor concentration was 10 µM. All compounds were screened against K667Δpdr5[PfATP6], along with two control strains K667Δpdr5[SERCA1a] and K667Δpdr5[pUG]. Means are of 2 biological replicates and 5 technical replicates for thaperoxides (TO), or 1 biological replicate for Saikosaponin D and *tert*-butyl peroxide. Where no obvious inhibition was observed further replicates were not performed. Saikosaponin D was only screened with K667Δpdr5[PfATP6], and *tert*-butyl peroxide was screened with K667Δpdr5[PfATP6] and K667Δpdr5[SERCA1a]. Any significant inhibition presented is the result of a false hit. **B.** Comparison of growth of yeast over time of the fluorescent (dashed line) versus non-fluorescent (solid line) yeast strains K667Δpdr5[PfATP6]$^{S769}$ and K667Δpdr5[Venus][PfATP6]$^{S769}$, and K667Δpdr5[PfATP6]$^{769N}$ and K667Δpdr5[mCherry][PfATP6]$^{769N}$. Means are of 2 biological replicates and 5 technical replicates ± S.D. Grown in YPD medium with no supplemental calcium **C.** *In vitro*

activity of PfATP6 (membrane-bound) with 1 μM free calcium, in presence of potent inhibitor DHA (hashed), less potent inhibitor CPA (white), and negative control deoxyartemisinin (black), normalised against the no drug control. No significant difference between any condition or concentration tested. Means are of 3 technical replicates ± S.D. **D.** *In vitro* activity of SERCA1a (membrane-bound) with 1 μM free calcium, in presence of 100 μM CPA. Means are of 3 technical replicates ± S.D. (** = $p < 0.005$).
